# Cancer therapy in a microbial bottle: Uncorking the novel biology of the protozoan *Toxoplasma gondii*

**DOI:** 10.1371/journal.ppat.1006523

**Published:** 2017-09-14

**Authors:** Barbara A. Fox, Kiah L. Butler, Rebekah B. Guevara, David J. Bzik

**Affiliations:** Department of Microbiology and Immunology, Geisel School of Medicine at Dartmouth, Lebanon, New Hampshire, United States of America; University of Wisconsin Medical School, UNITED STATES

Cancers emerge after the immune system fails to control and contain tumors. Multiple tumor-specific mechanisms create tumor environments where the immune system is forced to tolerate tumors and their cells instead of eliminating them. The goal of cancer immunotherapy is to rescue the immune system’s natural ability to eliminate tumors [1]. Here, we uncork the unique biology of the bottle-shaped protozoan *Toxoplasma gondii*. We reveal how this microbe when engineered into a safe nonreplicating vaccine effectively breaks tumor control over the immune system, which then unleashes potent immunity against already established cancer, thereby promoting survival and preventing cancer recurrence.

## Is cancer a disease of the immune system?

Cancer originates when normal cells are transformed into cells that do not stop replicating. Usually, the immune system recognizes transformed cells and eliminates them to prevent the development of cancer. Occasionally, however, environments develop that make this tumor elimination less effective. More than a decade ago, cancer experiments unexpectedly demonstrated that compared to healthy mice with intact immune systems, mice that lack intact immune systems develop more highly immunogenic tumors [2]. These findings led to the evolution of new concepts of cancer immunosurveillance and immunoediting [3]. The immune system suppresses cancer development by halting the replication of tumor cells and by killing tumor cells. However, this constant immune attack of the tumor also triggers adaptations by tumor cells that accelerate chronic inflammation at tumor sites and induces the remodeling of tissue (stroma), making it harder for new immune cells to gain access to the tumor environment. Ultimately, immune cells in the tumor environment adopt a more tolerogenic phenotype [4]. This tolerogenic phenotype, also called tumor immune tolerance, disarms the tumor-killing potential of immune cells and promotes cancer development. Cancer can thus be viewed as a chronic inflammatory disease of the immune system at tumor sites.

## Are cancer, inflammation, and microbial infection interrelated?

Many cancers have been linked with the presence of chronic inflammation associated with particular viral, parasitic, or bacterial infectious agents [5]. Tumor-promoting chronic inflammation is typically biased towards the T_H_2 immune response, which favors humoral immunity over cellular immunity (reviewed in reference [6]). In contrast, acute inflammation from the cellular T_H_1 immune response is strongly associated with increased cancer survival rates [7]. The antitumor effects of acute inflammation arise primarily from CD8^+^ T cells that selectively recognize and kill tumor cells [8]. Acute inflammation associated with infection has been observationally linked with the spontaneous elimination of tumors for centuries [9]. More than 1 hundred years ago, this association led William Coley to test the use of bacterial toxins as a cancer immunotherapy treatment, resulting in triggered tumor regression in certain tumor types. The molecular basis of Coley's toxin is the T_H_1 immune response and CD8^+^ T-cell immunity [10]. Acute inflammation induced by a variety of bacteria is associated with the spontaneous regression of tumors [11]. Additionally, commensal bacteria in the gut can also modulate inflammation at tumor sites [12].

Less is currently known about the antitumor effects of acute inflammation induced by unicellular eukaryotic protozoans. Decades ago, protein extracts from the protozoan *T*. *gondii* (hereafter, *Toxoplasma*) were reported to elicit partial antitumor effects (reviewed in reference [13]). In addition, *Toxoplasma* infection elicited detectable antitumor responses against B16F10 melanoma development by suppressing angiogenesis [14]. While it was known that *Toxoplasma* infection strongly induces a T_H_1-biased immune response [15], safe, nonreverting, and nonpathogenic strains of *Toxoplasma* were not previously available.

## Can nonreplicating *Toxoplasma* be used as a safe vaccine for cancer therapy?

**Fig 1:**
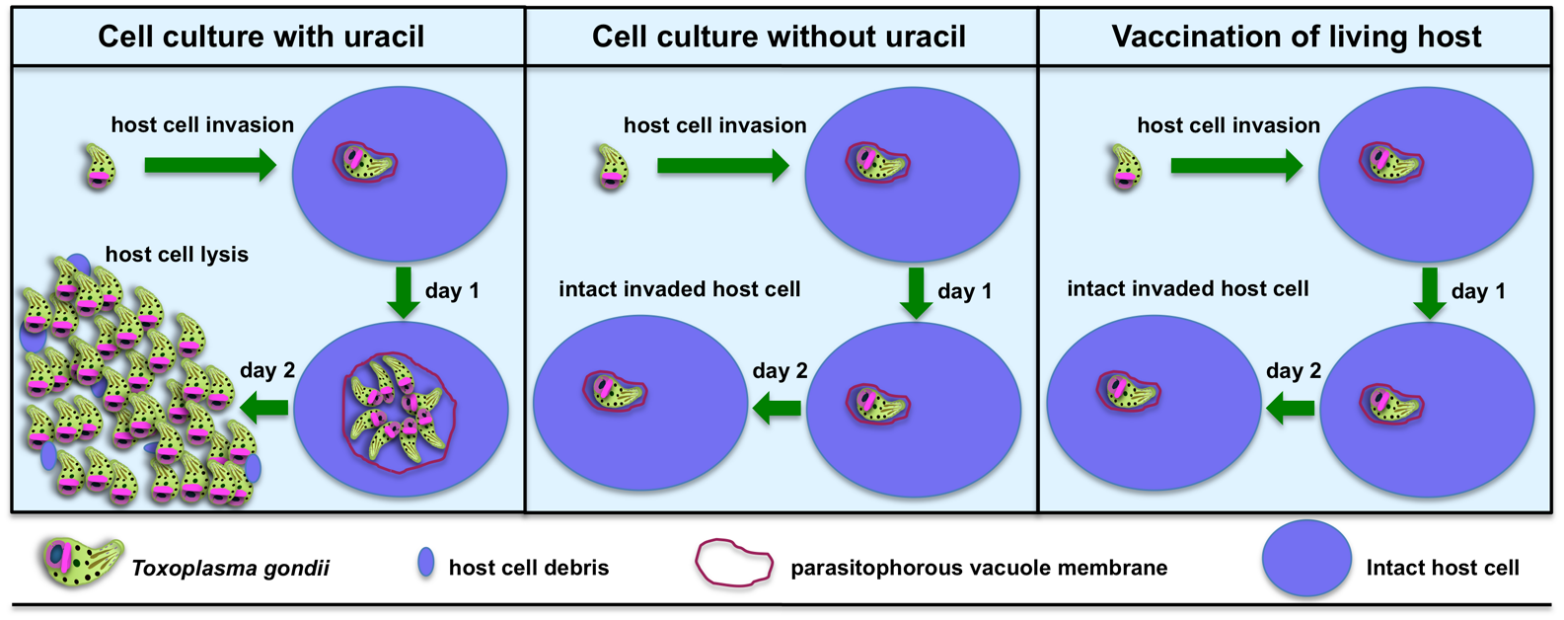
Nonreplicating *Toxoplasma* uracil auxotrophs (NRTUAs) do not replicate in living host animals. Left panel: *Toxoplasma* NRTUAs invade host cells in vitro and replicate normally if the nutrient uracil is added to the culture medium. Center panel: *Toxoplasma* NRTUAs invade host cells in vitro but do not replicate in the absence of uracil supplementation. Right panel: Mammalian hosts have extremely low uracil concentrations because they do not express the uracil phosphoribosyltransferase enzyme, and therefore, pyrimidine salvage instead occurs through nucleoside kinases that salvage the nucleoside uridine into uridine 5′-monophosphate (UMP) [17]. In living hosts, *Toxoplasma* NRTUAs invade host cells but do not replicate because there is insufficient uracil to support replication.

*Toxoplasma* is an obligate intracellular organism that replicates only after it has invaded a host cell [16]. *Toxoplasma* replication can be completely prevented by blocking the de novo pyrimidine synthesis pathway to halt the synthesis of uridine 5′-monophosphate (UMP), which is needed for RNA and DNA synthesis. This renders *Toxoplasma* into a uracil auxotroph since *Toxoplasma* also expresses a uracil phosphoribosyltransferase enzyme that can salvage exogenously supplied uracil directly into UMP to bypass any genetically induced block in de novo pyrimidine synthesis [17, 18]. Thus, genetically defined nonreverting uracil auxotrophs can easily be cultured in the laboratory in host cells that are fed with the nutrient uracil (Fig 1, left panel). In the absence of uracil, uracil auxotrophs invade host cells to form parasitophorous vacuoles but do not replicate (Fig 1, middle panel). In animals, uracil is not available, and uracil auxotrophs invade host cells but do not replicate (Fig 1, right panel).

Nonreplicating *Toxoplasma* uracil auxotrophs (NRTUAs) lack virulence in mice, and the immune system quickly clears the NRTUAs within approximately 5 days [19, 20]. NRTUAs are nonpathogenic and are safely tolerated even by severely immunodeficient animals that do not produce interferon gamma (IFN-γ) [17, 18] or that lack T cells, B cells, and natural killer cells (NOD/scid/gamma mice) [21]. Liver creatinine levels as well as alanine aminotransferase and aspartate aminotransferase levels were not affected in NOD/scid/gamma mice vaccinated with NRTUAs, further suggesting that NRTUAs are not toxic in mice [21]. In addition, while many toxic molecules have been described in various pathogenic bacteria, toxic molecules have not been reported for *Toxoplasma*. Moreover, a single low-dose vaccination with NRTUAs rapidly elicits a lifelong CD8^+^ T-cell-dependent protective immunity against reinfection by pathogenic strains of *Toxoplasma* [17, 18, 22, 23]. Remarkably, NRTUAs can elicit protective CD8^+^ T-cell immunity in mice after a single vaccination with only 10,000 nonreplicating organisms [24], a much lower vaccine dose than is required to elicit immunity induced by existing nonreplicating bacterial or viral vaccine platforms. These potent vaccine efficacy and strong safety profiles motivated us to test the potential of NRTUAs for cancer therapy. Lethal aggressive tumors were implanted in mice and allowed to develop for 7–12 days. Tumor-bearing animals were then vaccinated with NRTUAs. One hundred percent of ID8-Vegf ovarian cancer-bearing mice [25], 90% of B16F10 melanoma-bearing mice [21], and 40% of disseminated pancreatic (Pan02) tumor-bearing mice [26] survived these cancers without recurrence of the tumors. These were the first reports of long-term survival in mice bearing B16F10 melanoma or ID8-Vegf tumors after treatment with a single immunotherapeutic agent. Remarkably, the mice that survived these cancers also effectively resisted new tumors that were experimentally implanted [21, 26].

## How do NRTUAs trigger effective antitumor immunity?

NRTUA treatment of tumor-bearing mice at tumor sites rapidly increased the production of T_H_1 cytokines interleukin 12 (IL-12) and IFN-γ and activated tumor-associated and splenic CD8^+^ and CD4^+^ T cells [21, 25, 27]. Systemic NRTUA treatment also provoked a significant antitumor response in mice bearing aggressive ovarian tumors [20]. The depletion of T_H_1 cytokines IL-12 or IFN-γ or depletion of CD8^+^ T cells abolished the antitumor response to B16F10 melanoma [21], to ovarian cancer [20], and to pancreatic cancer [27]. Interestingly, *MyD88*-independent IL-12 production was sufficient for the antitumor response in the B16F10 melanoma and ovarian cancer models [20, 21], but not for effective therapy in the pancreatic cancer model [27]. In addition, NRTUA treatment rapidly promoted production of the T-cell-recruiting chemokines CXCL9 and CXCL10 in the tumor environment [21]. Moreover, analysis of the CD8^+^ T-cell populations after NRTUA treatment revealed dramatic increases in activated tumor antigen-specific CD8^+^ T cells that could specifically recognize and kill tumor cells. T_H_17 responses were not associated with the NRTUA-triggered antitumor responses [21, 25].

In live hosts, *Toxoplasma* preferentially invades innate myeloid cell types such as dendritic cells and monocytes/macrophages [28] to enable resistance to clearance by IFN-γ-dependent mechanisms [29]. Manipulation of myeloid cells by *Toxoplasma* invasion is crucial for the regulation of host immune responses, prevention of clearance, prevention of host death, and the establishment of chronic infection [30]. This same preferential targeting of myeloid cells for invasion is also observed after vaccination with NRTUAs [19, 25, 27]. Surprisingly, myeloid cells actively invaded by NRTUAs exhibit amazing signs of immune activation instead of immune suppression. Myeloid cell expression of costimulatory molecules CD80 and CD86, as well as major histocompatibility antigen I (MHCI), was markedly increased in NRTUA-invaded cells [19, 25, 27]. NRTUAs preferentially invade immunosuppressive tumor-associated dendritic cells, reverse their immunosuppressive phenotype to an immune-activated phenotype, and rescue their ability to process and present tumor antigens to activate tumor antigen-specific CD8^+^ T cells [25]. CD11c^+^CD8α^+^ dendritic cells cross present tumor antigens in the context of MHCI and costimulatory molecules to potently activate CD8^+^ T-cell immunity to cancer [31]. Tumor-bearing *Batf3*^-/-^ knockout mice deficient in CD11c^+^CD8α^+^ dendritic cells failed to mount any antitumor response after NRTUA treatment [20]. Altogether, these results suggest that NRTUAs targeted the invasion of dendritic and other myeloid cells in the immunosuppressive tumor environments to trigger mechanisms that break tumor immune tolerance and awaken potent CD8^+^ T-cell immunity to established cancers.

## What's in the *Toxoplasma* microbial bottle that triggers immunity to cancer?

**Fig 2:**
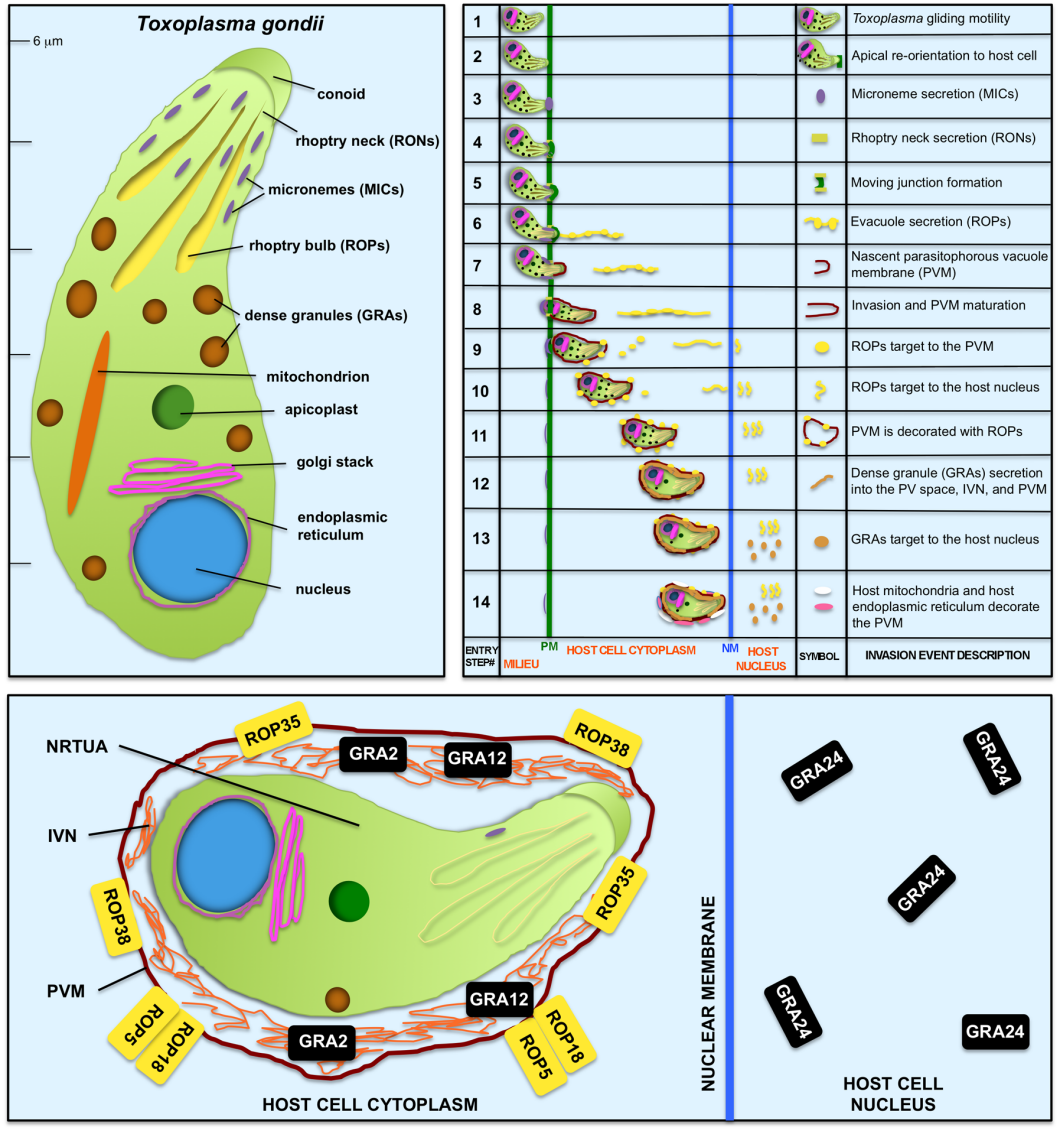
*Toxoplasma* is a bottle-shaped eukaryotic microbe that uses specialized secretory organelles to invade the host cell, to establish a parasitophorous vacuole, and to manipulate host signaling and transcriptional pathways. Top-left panel: *Toxoplasma* secretory organelles. Top-right panel: 14 events that occur during invasion of the host cell to establish the parasitophorous vacuole habitat as well as manipulation of the host cell (adapted from reference [16]). The displayed order of these invasion steps should be interpreted cautiously at this time since the elucidation of the precise order of some of these steps and the mechanisms regulating gliding motility, invasion, and secretion events are major topics of ongoing research into the biology of *Toxoplasma* [16, 29, 32]. Bottom panel: An expanded view of an NRTUA-invaded cell shows the host cell localization of *Toxoplasma* secreted effectors necessary to trigger an antitumor response. See the main text for explanations. Abbreviations: GRA, protein secreted from the dense granules; IVN, intravacuolar network; MIC, microneme adhesin or other microneme secreted protein; NM, nuclear membrane of the host cell; NRTUA, nonreplicating *Toxoplasma* uracil auxotroph; PM, plasma membrane of the host cell; PVM, parasitophorous vacuole membrane; PV space, parasitophorous vacuole space; RON, rhoptry neck secreted protein; ROP, rhoptry bulb secreted protein.

*Toxoplasma* is an approximately 6-μm-long bottle-shaped eukaryotic microbe with specialized secretory organelles called micronemes, rhoptries, and dense granules (Fig 2, left panel). *Toxoplasma* gliding motility and regulated secretion from these specialized secretory compartments mediate invasion of the host cell as well as the development of the parasitophorous vacuole membrane (PVM) compartment that surrounds intracellular *Toxoplasma* (Fig 2, right panel) [16]. The secretion of rhoptry (ROP) effectors [29] and dense granule (GRA) effectors [32] during invasion also provides a molecular basis for manipulation of host cell signaling and transcriptional pathways.

Is NRTUA invasion of host cells required for the antitumor response? Yes. Treating tumors with secreted *Toxoplasma* molecules, whole *Toxoplasma* cell extracts, or noninvasive NRTUAs failed to trigger any antitumor response [20]. While noninvasive NRTUAs still triggered IL-12 production, the production of IFN-γ was not triggered, showing that active invasion of host cells by NRTUAs was critical for T-cell production of IFN-γ and the antitumor response. Is secretion of *Toxoplasma* effectors required for the antitumor response? Yes. Blocking secretion of ROP proteins using 4-Bromophenacyl Bromide [20] prevents secretion and invasion and completely abolishes the antitumor response. Blocking gliding motility using mycalolide B to prevent *Toxoplasma* invasion without blocking the secretion of ROP effectors into host cells (Fig 2, top-right panel) weakened the antitumor responses [20]. Thus, NRTUA invasion and secretion of specific effectors, and perhaps secretion of GRA effectors, appeared to be essential for antitumor immunity.

To test this hypothesis, genes encoding a number of ROP and GRA parasite secreted effectors were deleted, and the antitumor response triggered by these NRTUA mutants was measured [20]. While the deletion of several secreted effector molecules did not influence the antitumor response, the deletion of ROP5, ROP18, ROP35, ROP38, GRA2, GRA12, or GRA24 markedly reduced the antitumor responses [20]. ROP5, ROP18, ROP35, and ROP38 are associated with the host cytosolic face of the PVM (Fig 2, bottom panel) [20]. GRA2 and GRA12 occupy a vacuole tubulovesicular membrane system, called the intravacuolar network (IVN), which links *Toxoplasma* to the PVM (Fig 2, bottom panel) [33]. GRA24 is one of the GRA proteins that reaches the host cell nucleus (Fig 2, bottom panel) to modulate host cell signaling and transcription [32]. GRA24 bypasses the classical mitogen-activated protein kinase phosphorylation mechanism to induce a sustained autophosphorylation of host cell p38α to activate downstream transcription factors [34]. While the roles of ROP35 and ROP38 remain to be determined, the ROP5/ROP18 PVM-associated protein complex phosphorylates host p47 immunity-related GTPases to neutralize IFN-γ-dependent host mechanisms that clear *Toxoplasma* vacuoles (reviewed in reference [29]). Surprisingly, while the PVM association of ROP18 was required, the kinase function of the ROP18 complex was not required to trigger the antitumor response [20]. Consistent with these findings, NRTUAs generated from type II *Toxoplasma* strains were as effective in cancer therapy as NRTUAs generated from type I *Toxoplasma* strains [20]. Mechanistically, these genetic studies suggested that it is the active modulation of NRTUA-invaded host cell signaling and transcriptional pathways by ROP effectors and GRA effectors secreted into immunosuppressive myeloid cells in the tumor environment, in conjunction with other host responses triggered by the presence of NRTUAs, that not only rescues the processing and presentation of tumor antigens to activate tumor antigen-specific CD8^+^ T cells but also breaks tumor immune tolerance to awaken the potent tumor cell-killing functions of the activated tumor antigen-specific CD8^+^ T-cell populations.

## Conclusions

Questions still remain to be answered regarding the mechanisms triggered by NRTUAs and their novel secreted molecules that successfully modulate tumor-associated dendritic and other myeloid cells to effectively break tumor immune tolerance. NRTUA invasion and secretion of effector molecules regulate antigen presentation to drive highly effective CD8^+^ T-cell immunity. Thus, NRTUAs in essence behave as a safe and potent intracellular T_H_1 adjuvant for dendritic cells. Since NRTUAs are easily genetically engineered to express heterologous antigens that are vigorously presented by MHCI to activate antigen-specific CD8^+^ T cells [18, 23], NRTUAs represent a novel and broadly applicable T_H_1 vaccine platform with the ability to elicit potent CD8^+^ T-cell-dependent protective immune responses not only against cancer but perhaps also against various intracellular protozoan (malaria), bacterial (tuberculosis), or viral (HIV) pathogens where a more effective T_H_1 cellular immunity could be beneficial to prevent infection or eradicate existing infection. NRTUAs safely triggered effective antitumor immunity regardless of whether the tumor-bearing animals were immune to *Toxoplasma* prior to NRTUA treatment [20]. Thus, in contrast to existing bacterial and viral anticancer platforms that are rendered less effective by immunity [35], pre-existing immunity to *Toxoplasma* is not a barrier to this potential cancer therapy. These remarkable efficacy and strong safety profiles suggest that NRTUAs may trigger immunity to cancer in humans, though further work is needed to evaluate NRTUA safety and efficacy in human tumors. In addition, the NRTUA-triggered mechanisms that awaken the tumor-killing functions of tumor antigen-specific CD8^+^ T cells are important to characterize since these killing functions are commonly shut down in immunosuppressive tumor environments. Further investigation of the NRTUA-elicited antitumor mechanisms could reveal the specific targets in mammalian cell signaling and transcriptional pathways that can be manipulated to break tumor immune tolerance and rescue natural and effective CD8^+^ T-cell immunity to cancer.
